# Metal/Carbon Hybrid Nanostructures Produced from Plasma-Enhanced Chemical Vapor Deposition over Nafion-Supported Electrochemically Deposited Cobalt Nanoparticles

**DOI:** 10.3390/ma11050687

**Published:** 2018-04-27

**Authors:** Mohammad Islam, Amine Achour, Khalid Saeed, Mohammed Boujtita, Sofia Javed, Mohamed Abdou Djouadi

**Affiliations:** 1 Center of Excellence for Research in Engineering Materials, Deanship of Scientific Research, King Saud University, P.O. Box 800, Riyadh 11421, Saudi Arabia; 2 LISE Laboratory, Research Centre in Physics of Matter and Radiation (PMR), University of Namur, B-5000 Namur, Belgium; a_aminph@yahoo.fr; 3 Department of Mechanical Engineering, College of Engineering, King Saud University, P.O. Box 800, Riyadh 11421, Saudi Arabia; khalid.uetp@gmail.com; 4 CEISAM: Chimie et Interdisciplinarité: Synthèse Analyse Modélisation, UMR 6230 CNRS—Université de Nantes, UFR Sciences et Techniques, Nantes CEDEX 3, France; Mohammed.Boujtita@univ-nantes.fr; 5 School of Chemical and Materials Engineering, National University of Sciences & Technology, Islamabad, Sector H-12, Islamabad 44000, Pakistan; sofia.javed@scme.nust.edu.pk; 6 Institut des Matériaux Jean Rouxel, UMR 6502, 2 rue de la Houssinière, B.P. 32229, F-44322, Nantes CEDEX 3, France; Abdou.Djouadi@cnrs-imn.fr

**Keywords:** cobalt nanoparticles, carbon nanotubes, Nafion membranes, nanocomposite films, electrochemical process, AFM

## Abstract

In this work, we report development of hybrid nanostructures of metal nanoparticles (NP) and carbon nanostructures with strong potential for catalysis, sensing, and energy applications. First, the etched silicon wafer substrates were passivated for subsequent electrochemical (EC) processing through grafting of nitro phenyl groups using para-nitrobenzene diazonium (PNBT). The X-ray photoelectron spectroscope (XPS) and atomic force microscope (AFM) studies confirmed presence of few layers. Cobalt-based nanoparticles were produced over dip or spin coated Nafion films under different EC reduction conditions, namely CoSO_4_ salt concentration (0.1 M, 1 mM), reduction time (5, 20 s), and indirect or direct EC reduction route. Extensive AFM examination revealed NP formation with different attributes (size, distribution) depending on electrochemistry conditions. While relatively large NP with >100 nm size and bimodal distribution were obtained after 20 s EC reduction in H_3_BO_3_ following Co^2+^ ion uptake, ultrafine NP (<10 nm) could be produced from EC reduction in CoSO_4_ and H_3_BO_3_ mixed solution with some tendency to form oxides. Different carbon nanostructures including few-walled or multiwalled carbon nanotubes (CNT) and carbon nanosheets were grown in a C_2_H_2_/NH_3_ plasma using the plasma-enhanced chemical vapor deposition technique. The devised processing routes enable size controlled synthesis of cobalt nanoparticles and metal/carbon hybrid nanostructures with unique microstructural features.

## 1. Introduction

Nanoparticles (NP) synthesis with precise control is a major challenge in the continuously evolving field of nanotechnology for applications in the biomedicine, energy, chemical, and manufacturing industries. Although various techniques such as mechanical grinding, polyol process, organometallic precursor pyrolysis, alcohol reduction, microemulsion technique, and template-assisted growth can be employed to produce ultrafine NP with narrow size distribution [1,2,3,4,5,6], electrochemical (EC) reduction offers two distinct advantages over other processes: (i) NP with desirable size and distribution can be obtained by tuning the process parameters, thus eliminating the need for polymeric additives to inhibit growth of nuclei formed, and (ii) surface-supported NP are obtained in-situ in contrast with colloidal route where the NP need to be re-dispersed on a surface after synthesis.

For NP synthesis over silicon substrate, the electrical conductivity of the surface must be preserved. After removal of the native oxide layer, the free aryl radicals can be electrochemically produced that bond with silicon, resulting in the formation of mono- and multi-layers [7]. Electrochemical grafting can be carried out in aprotic as well as aqueous acidic media and has strong implications for use in many biological and electronic applications [8,9,10,11]. Unlike self-assembled monolayers, direct coupling of organic molecules with a surface involves formation of a strong covalent bond [12]. The EC reduction of aryl diazonium salt on silicon surface causes generation of aryl radical (along with N_2_ molecule) followed by hydrogen abstraction from the surface by the aryl radical and subsequent formation of a ≡Si–Ph bond. Whether these processes occur step-by-step or simultaneously depends on the solution chemistry and grafting conditions [7]. Formation of a monolayer or multilayers is possible through control of electrochemical reduction parameters such as solution chemistry, applied potential, and time. It is noteworthy that conditions to obtain monolayer vary for different substrate types [13].

Nafion membranes are copolymers of tetrafluoroethylene and perfluorinated vinyl ether containing terminated sulfonyl fluoride group. In a salt bath solution, the cations tend to cluster in the polymer-solution phase and may or may not invade into the membrane depending on the salt concentration [14]. The subsequent EC reduction leads to the formation of nanoparticles the average size of which depends on salt bath composition and electrochemical conditions. Composite coatings of Nafion with ultrafine nanoparticles offer a novel route towards enhancement of thermal, chemical, and electrical properties of pristine Nafion membranes. Yoon et al. [15] reported synthesis of homogeneously dispersed, non-agglomerated nickel nanoparticles with radii in the range of 1.5–3 nm in the Nafion membrane. Incorporation of cobalt porphyrin (CoP) complex in the hydrophobic domain of Nafion was found to facilitate the oxygen transport through the membrane, thus promising an increase in oxygen consumption efficiency for fuel cell applications [16]. Sode et al. [17] found that pre-soaking of Nafion membranes in Pt salt precursor solution followed by reduction in NaBH_4_ produced platinum nanoparticles to a certain distance from the Nafion membrane surface, depending on the state of hydration and pH of the soaking solution. Addition of inorganic metal oxide nanoparticles into Nafion membrane was explored to investigate charge transport properties in proton exchange membrane fuel cells [18,19,20,21] and waste water treatment for environmental remediation [22,23].

In this paper, we report size controlled synthesis of cobalt nanoparticles over Nafion membranes and subsequent growth of carbon nanostructures via plasma-enhanced chemical vapor deposition (PECVD) process. Using silicon wafer as a substrate, the native oxide layer was first removed, followed by electrochemical grafting of para-nitrobenzene diazonium (PNBD) salt to preserve the conductive nature of the substrate. The effects of Nafion solution, the deposition process, and deposition conditions on the thickness and morphology of the membrane films produced via the dip or spin coating process were investigated. The electrochemical reduction conditions were varied to assess their effect on NP size and distribution. Low-pressure, microwave plasma enhanced chemical vapor deposition process was employed to grow carbon based nanostructures.

## 2. Results and Discussion

### 2.1. Aryl Diazonium Grafting and Nafion Membranes Deposition

**Figure 1:**
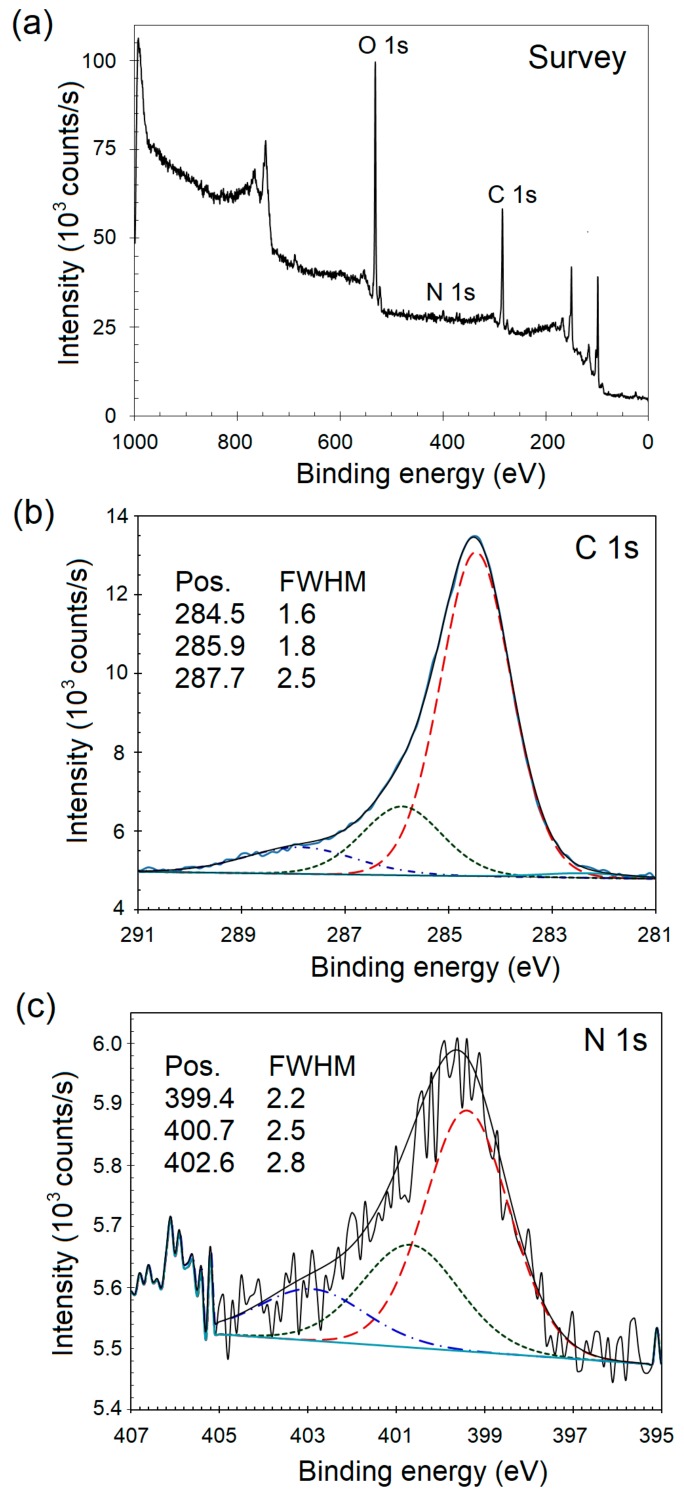
(**a**) XPS survey spectrum of the electrochemically grafted para-nitrobenzene diazonium (PNBD) and high resolution valence band regions with component peaks for (**b**) the C 1s and (**c**) the N 1s envelopes.

Immediately after electrochemical grafting with diazonium (PNBD) salt and prior to NP synthesis, the sample S_1_ was transferred to the chamber for XPS analysis. The survey spectrum as well as high resolution C 1s and N 1s valence band regions are presented in Figure 1. From Figure 1a, the peaks characteristic of the C 1s, N 1s, and O 1s can be readily identified, although the intensity of the N 1s peak is low. The main C 1s peak was found to be asymmetric and centered at ~284.6 eV. The high resolution C 1s peak (Figure 1b) was deconvoluted into one very strong peak and two relatively less strong, broad, shoulder peaks in the 286–288 eV range that may be attributed to the oxidized carbon atoms [24]. The C 1s spectrum was curve-fitted using component peaks at 284.5, 285.9, and 287.7 eV with respective assignments to the C–C, C–O, and C=O groups [25,26]. For comparison of two or more samples, the full-width at half-maximum (FWHM) values quantify the peak broadening and indicate one or more of these: (i) any changes in the number of chemical bonds responsible for that particular peak, (ii) a possible change in the sample condition e.g., upon radiation damage, and (iii) localized differences in the surface charge states. In this case, however, XPS analysis was performed on only one sample to investigate surface composition and the effect of EC grafting on the etched Si substrate. The N 1s core level shown in Figure 1c is believed to have formed from component peaks at 399.4, 400.7, and 402.6 eV. The peak at 399.4 maybe assigned to the amine group (C–NR_2_; R=C, H). Another peak centered at 400.7 eV can be attributed to NH_2_ terminal group and is referred to as pyrrolic N (sp^2^ C–N bonding) where a nitrogen atom is bonded to two carbon and one hydrogen atoms. The 402.6 eV peak can be assigned to the terminal protonated amines (NH_3_^+^) and its presence is also indicative of multilayer formation. Although not labeled, the weak peak located at 406 eV is assigned to the formation of nitro groups (–NO_2_). Generally, the binding energies in the range of 402 to 410 eV represent oxidized N [27,28,29,30]. The FWHM values for the component peaks after deconvolution of the C 1s and N 1s core level peaks are listed in Figure 1.

**Figure 2:**
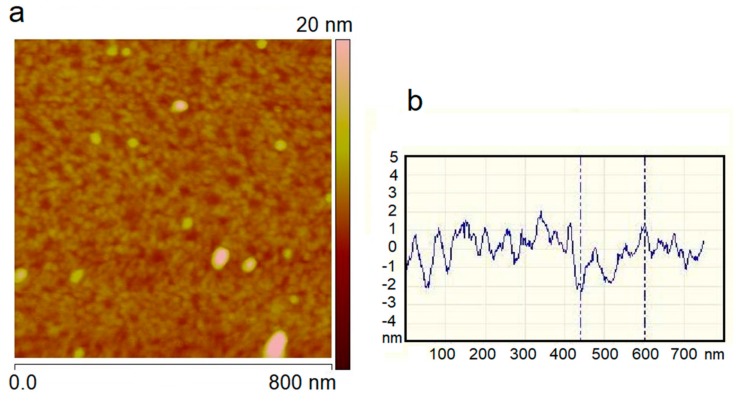
Atomic force microscope (AFM) results of the aryl grafted silicon surface after electrochemical reduction in the diazonium salt and sulfuric acid mixed solution (S_1_, Table 1): (**a**) Two-dimensional area scan and (**b**) the line profile showing vertical displacement versus distance.

The 2-dimensional area scan of the surface microstructure and the corresponding line profile for the sample S_1_ are shown in Figure 2. The features with relatively larger vertical height represent nanoparticles that were subsequently electrodeposited over aryl diazonium layer and will not be discussed here. The 0.8 µm area scan reveals a blocking layer formation due to reaction between the silicon surface electrode and the nitrophenyl radicals generated from reduction of the diazonium moiety [31]. The peak-to-peak roughness values from the line scan were found to be in multiples of 0.7 nm, which is in accordance with the reported value for grafted PNBD monolayer [7]. The line profile exhibited a maximum step height of <5 nm, implying formation of up to 6 or 7 layers on the etched silicon surface. The results suggest that although the grafted layer exhibited thickness variation over the substrate surface, the electrochemical reduction conditions employed ensured passivation of the silicon surface for subsequent processing of the sample. Thus, constant potential amperometry at a very negative potential for −1.5 V for a very brief period of 150 ms passivates the etched silicon surface through an organic film grafting for subsequent NP synthesis procedure.

The samples S_2_–S_4_ (Table 1) were produced to assess the effect of Nafion solution composition and the film deposition on thickness and quality of the resulting membranes. For that purpose, the Nafion films were produced over freshly exposed Si substrates after HF etch via dip coating or spin coating process. It was observed that 0.5 wt % Nafion precursor solution yielded membranes with greater thickness in case of both dip and spin coating processes. The membrane thickness was found to be up to 100 nm, depending on the process and spin speed (Supplementary Materials, Figure S1). The membrane characteristics in terms of thickness uniformity, surface roughness, and quality (pin-hole defects) were noticed to be strongly influenced by the deposition technique. The spin coated films were more smooth and defect-free than those produced using dip coating technique. The values of membrane thickness after deposition under different conditions are listed in Table 1. The Nafion concentrations of 0.5 and 1.0 wt % affect film thickness after spin coating process with respective values of 180 ± 11 and 87 ± 5.1 nm. 

### 2.2. NP Synthesis: Effect of Nafion Deposition Process and Electrochemistry Parameters

#### 2.2.1. Dip Coating Versus Spin Coating Process

**Figure 3:**
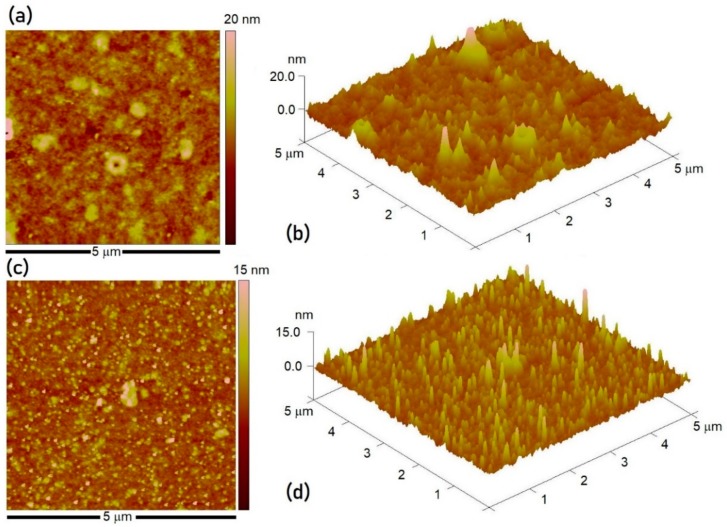
2-D and 3-D AFM scans showcasing the effect of Nafion membrane deposition process on the film morphology and the NP size and distribution after indirect EC reduction for the (**a**,**b**) dip coating (sample S_2_) and (**c**,**d**) spin coating (sample S_3_) processes.

The Nafion membranes were initially immersed in the 0.1 M CoSO_4_ solution for 3 h in order to ensure significant uptake of Co^2+^ ions for subsequent electrochemical reduction in 0.5 M H_3_BO_3_ solution at −1.5 V for 5 s. The effect of the Nafion film deposition process on the resulting film morphology, nanoparticle size, and distribution was compared in case of S_2_ and S_3_ samples using 2-dimensional AFM scans as well as 3-dimensional topography of the Nafion films (Figure 3). While both the surfaces revealed formation of very fine nanoparticles, the Nafion membranes obtained from dip coating process, however, were not defect-free and exhibited presence of surface pores and blow-holes. On the other hand, Nafion membranes prepared using the spin coating process were smooth and of high quality with no defects. The difference in morphology may be attributed to the inherent characteristics of the two processes, since the spin coating technique produces films of relatively less thickness, greater degree of thickness uniformity, and better quality in terms of surface homogeneity and low defect density. From the AFM results, the average NP size estimated for the dip coated and spin coated membranes was 5.4 ± 1.6 and 4.8 ± 2.0 nm, respectively. Although the NP obtained were of the same size range regardless of the Nafion membrane deposition technique, the distribution of NP over the dip coated membrane was not uniform due to poor morphology. Nevertheless, both types of membranes yielded nanoparticles with ultrafine size and narrow size distribution. Thus, for a relatively short reduction time of 5 s, the thickness of Nafion membrane does not seem to have a significant influence on the average NP size.

#### 2.2.2. Indirect Versus Direct Electrochemical Reduction

**Figure 4:**
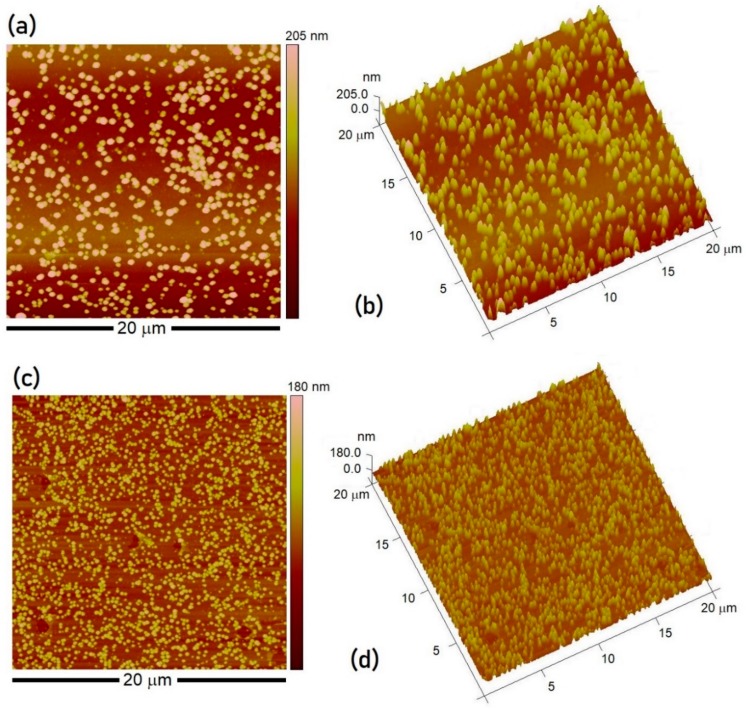
The 20 µm area scans showing AFM images and the 3-D topography of the Nafion supported Co-based nanoparticles after 20 s EC reduction (S_4_ vs. S_5_, Table 1): (**a**,**b**) Co^2+^ immersion followed by EC reduction (IR, S_4_) and (**c**,**d**) direct EC reduction in the CoSO_4_+H_3_BO_3_ solution (DR, S_5_).

Two samples (S_4_ and S_5_) were prepared under identical sets of processing steps with only one difference. While the CoSO_4_ solution concentration was kept the same (0.1 M CoSO_4_) in both cases, the sample S_4_ was pre-treated with CoSO_4_ solution for 3 h followed by EC reduction in 0.5 M H_3_BO_3_ solution at −1.5 V for 20 s. On the other hand, the sample S_5_ was obtained from simultaneous Co^2+^ uptake and electrolytic reduction in 0.1 M CoSO_4_ 0.5 H_3_BO_3_ mixed solution at −1.5 V for 20 s. The 20 μm area scans and the 3-dimensional topography of the microstructures for both samples are represented in Figure 4 to illustrate size and distribution of the NP produced. In case of two-step synthesis (S_4_ sample), the NP size distribution was noticed to be bimodal with average sizes of 5.3 ± 1.1 and 128 ± 12 nm. The relatively longer reduction time of 20 s, as compared to 5 s, was also found to adversely affect the particle distribution over the substrate besides a tendency of cluster formation at some regions with each cluster comprising of about 8 to 15 crystallites (Figure S2). It is believed that the degree of Co^2+^ ion impregnation into the Nafion membranes from 3 h immersion in salt solution is very high. Constant potential amperometry for longer times promotes nucleation process during the entire period of electrodeposition, thus causing a broad range in the NP size (by two orders of magnitude) and inhomogeneous NP distribution over the surface. In case of sample S_5_, electrochemical synthesis in presence of both metal ion precursor solution and the reducing agent accounted for the NP formation with an average size of 65.2 ± 12.6 nm (Figure 4c,d). This implies that a prior immersion into ion solution is not required as the Nafion membrane, owing to its fine porous structure, possesses high ion uptake capability. The direct electrochemical reduction also proves to be a more efficient, one-step route toward synthesis of more fine nanoparticles.

#### 2.2.3. Effect of Electrochemical Reduction Time

**Figure 5:**
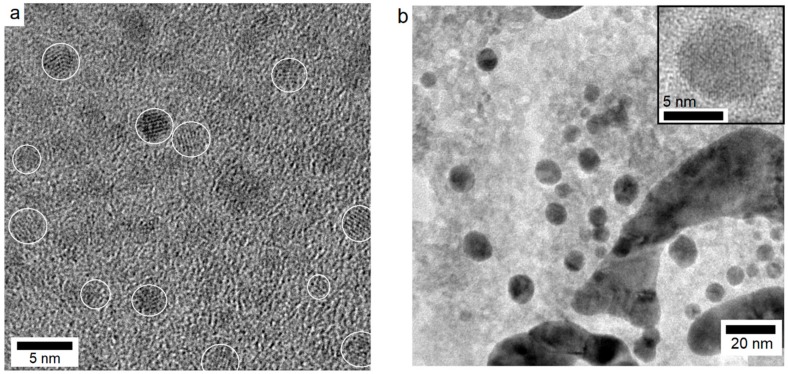
TEM microstructures of the samples S_6_ and S_7_ (Table 1) showing the NP size dependence on the EC reduction time in 1 mM CoSO_4_ 0.5 M H_3_BO_3_ solution: (**a**) 5 s and (**b**) 20 s.

Further experimentation was carried out by eliminating the CoSO_4_ solution impregnation step and instead, using a CoSO_4_ and H_3_BO_3_ solution mixture in 1:500 (mol/mol) ratio (1 mM CoSO_4_ 0.5 M H_3_BO_3_). The samples S_6_ and S_7_ were prepared with the intention to obtain very fine NP size via high nucleation rate induced by much greater H_3_BO_3_ concentration as reducing agent. The influence of EC reduction time was investigated by performing EC reduction for 5 and 20 s. The high-resolution TEM microstructures are presented in Figure 5, which show the nanoparticles to exhibit spherical morphology and distinctly different size range depending on the electrochemical reduction time. The inset in Figure 5b represents an individual cobalt nanoparticle with diffraction fringes characteristic of hexagonal cobalt phase with inter-planar spacing of 0.216 nm. From TEM observations, the average NP size for the S_6_ and S_7_ samples were estimated to be 2.8 ± 0.4 and 9.8 ± 2.6 nm, respectively. The inter-planar spacing (d_khl_) values for the different crystallites were found to be 0.191 and 0.204 nm for the hcp cobalt. The d_hkl_ values of 0.242 and 0.286 nm, however, were indexed to be (111) and (220) crystallographic planes from the CoO (JCPDS card no. 70-2855) and the face-centered cubic Co_3_O_4_ phases (JCPDS card no. 42-1467), respectively [32]. It is evident from the HR-TEM observations that the NP are present both as cobalt metal and cobalt oxides (CoO and Co_3_O_4_) indicating air oxidation of some of the Co NP. The fact that fine metal particles can easily get oxidized is also reported elsewhere [33,34]. These experiments demonstrate that direct electrochemical reduction of Nafion membranes produces NP with reduced size and more homogeneous dispersion within the Nafion membranes.

### 2.3. PECVD Growth of Carbon Nanostructures

**Figure 6:**
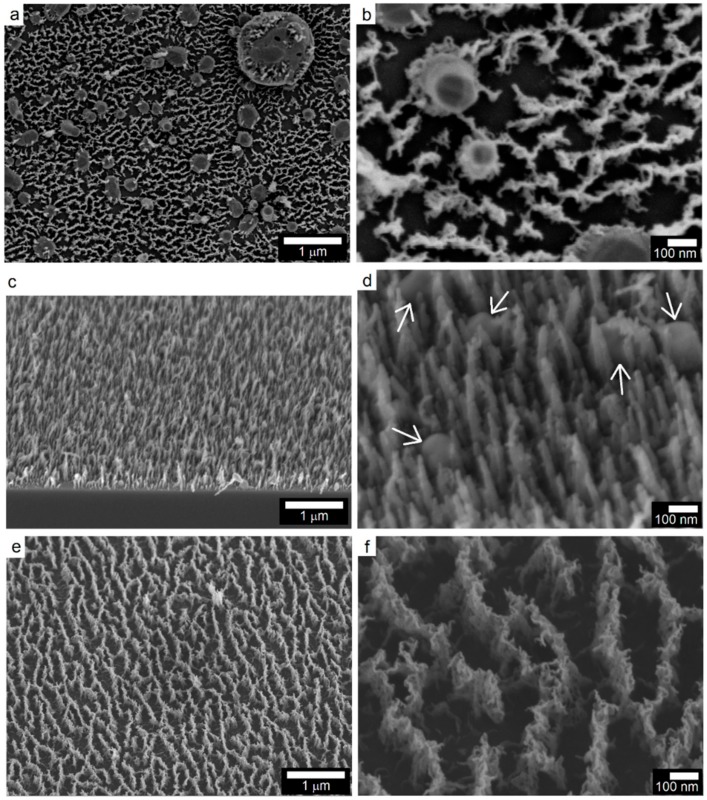
Low and high magnification SEM micrographs of the carbon nanostructures after PECVD growth over Si substrates supporting NP with different sizes: (**a**,**b**) Sample S_4_, (**c**,**d**) Sample S_5_, and (**e**,**f**) Sample S_7_.

The effect of catalyst NP size on the PECVD growth of carbon nanostructures was investigated by carrying out deposition over the samples S_4_, S_5_, and S_7_ under the same synthesis conditions. The electron microscopy of the surfaces at low and high magnification revealed interesting features, as presented in Figure 6. The initial ramping of the Si substrate containing metal/metal oxide NP to synthesis temperature of 900 °C caused an increase in the catalyst NP size via coalescence due to surface diffusion, thus leading to a relatively larger size (Figure 6a,b) than that determined after the NP synthesis (sample S_4_). In case of large nanoparticles, a composite surface of nanoparticles and vertically oriented carbon nanostructures was seen. The growth features were uniformly dispersed over the surface and, in some cases, appeared to be originating from the NP surface. At high magnification (Figure 6b), the carbon nanostructures were revealed to exhibit fibrous/tubular morphology with certain degree of entanglement and growth in a direction perpendicular to the substrate surface. The diameter and length of these nanostructures was estimated to be 9.1 ± 0.7 nm and a few tens of nanometers, respectively. Since the as-prepared NP were found to have bimodal size distribution, it is believed that fine nanoparticles catalyze nucleation and subsequent growth of short, thin tubular structures. The reduction of cobalt oxide to metallic Co. NP were also evident from slight difference in phase contrast between the NP core and its outer area and the observation that CNT growth appeared to have originated from the NP surface. The EDS elemental maps and the EDS spectrum confirmed both the uniform distribution of fine Co NP and their oxidized state (Figure S3). For the sample S_5_, the aligned carbon nanotubes (CNT) were produced from the PECVD process. From the high magnification view (Figure 6d), the average CNT diameter was computed to be 19 ± 3.7 nm. In addition to other CNT synthesis and growth conditions namely precursor gas composition and flow rate, temperature, and pressure, the catalyst NP size also plays an important role in defining the CNT outer diameter [35,36,37]. In addition to CNT, large nanoparticles with size in the range of 120 to 160 nm were also observed in the microstructure, as pointed out by white arrows. Under the identical PECVD conditions, the sample S_7_ yielded carbon nanostructures that were quite similar to those observed in the case of S_4_. The vertically oriented carbon nanostructures were reported to grow in presence of high density plasma under the strong influence of localized electric field and low gas pressure [38].

**Figure 7:**
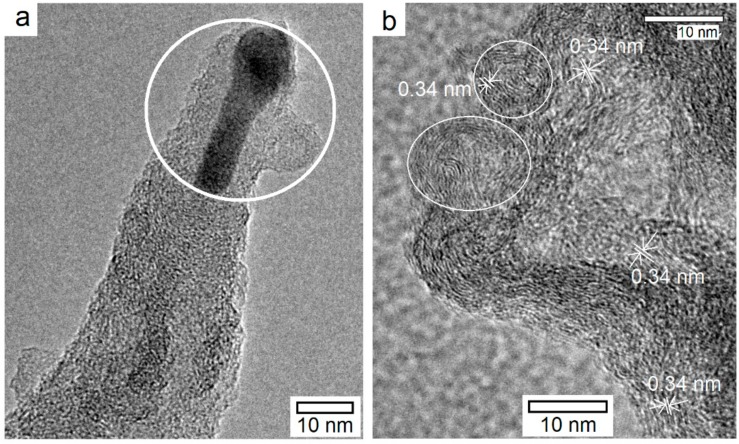
High-resolution TEM microstructures of the carbon nanostructures after PECVD at 900 °C using C_2_H_2_:NH_3_ mixture of 1:5.3 (sccm): (**a**) The near-tip area of an individual carbon nanofiber, (Sample S_5_) and (**b**) top view showing few-walled CNT with open ends and carbon nanosheets (Sample S_7_).

The high-resolution TEM examination of the samples S_5_ and S_1_, as demonstrated in Figure 7a revealed interesting microstructural features. In case of S_5_, the CNT exhibited tip-growth [39] as manifested by the presence of an elongated cobalt NP near the top end of an individual CNT. The particle appears to have undergone elongation upon softening/pre-melting at the synthesis temperature of 900 °C besides reduction by hydrogen. From absence of diffraction fringes from concentric graphitic shells and a hollow core typical of multiwalled carbon nanotubes, it is speculated that extensive lattice defects generation with associated significant loss of crystallinity occurred due to bombardment of N^3+^, H^+^ ions, and N_x_H_y_-based radicals that were generated in the ammonia-rich C_2_H_2_/NH_3_ plasma. Although the plasma pre-treatment power has been reported to induce a transition from tip-growth to base-growth mechanism [40], a fine catalyst size in our case resulted in base growth mechanism during nanotubes synthesis, as shown in Figure 7b. This is in agreement with a previous report, where a transition from tip- to the base-growth upon reduction in the catalyst NP size was reported [41]. In the case of sample S_1_, two individual nanotubes with open tips were evident, as indicated by white circles. Furthermore, the presence of carbon nanosheets was also depicted by the curved features that were assigned to the graphitic structure with a distance between successive planes to be 0.34 nm. The damage to the growing nanostructure, as induced by the plasma ions/radicals, was evident in terms of order disruption in the outermost layers besides presence of dangling bonds. The TEM findings nevertheless confirmed observations made during SEM examination of the samples. Size-controlled synthesis of cobalt and cobalt oxide nanoparticles over Nafion membranes and the Co–C based nanocomposites can be explored for diverse applications including proton exchange membrane fuel cells, catalysis, supercapacitors, sensors, and batteries [34,42,43,44,45,46,47].

## 3. Experimental

All reagents were used as received. 4-Nitrobenzenediazonium tetrafluoroborate (PNBD), sulfuric acid (98% H_2_SO_4_), hydrofluoric acid (HF 50% *w/w*), ethanol (98%), cobalt sulfate (CoSO_4_·7H_2_O), and boric acid (H_3_BO_3_) were procured from Sigma Aldrich. Highly-doped silicon (Ar^+^–Si n-type) coupons, 5 × 20 mm^2^ in size, were used as substrates for this work.

Immediately after etching in 1 wt % HF solution for 5 min for removal of native oxide layer, the substrates were transferred to the electrochemical cell for PNBD grafting. This was done to preserve conductive nature of the surface for electrochemical reduction of metal ions at the later stage. As opposed to potential cycling generally employed for reduction and subsequent grafting of diazonium salt, chronoamperometry experiments were performed by applying a potential of −1.2 V. The PNBD salt concentration used was 5 mM in 10 mM H_2_SO_4_ solution.

Subsequent to PNBD grafting, Nafion thin films were deposited via dip or spin coating using 0.5 or 1.0 wt % Nafion solution in ethanol. For this purpose, commercially available Nafion perfluorinated resin solution (CAS#31175-20-9; Sigma-Aldrich, Saint Louis, MO, USA) was used. The effect of precursor solution composition and spin coating speed on Nafion film thickness and nanoparticle characteristics were assessed.

For NP synthesis, two different approaches were adopted. Initially, Nafion coated samples were immersed into aqueous CoSO_4_ solution for 3 h for Co^2+^ ion uptake followed by electrochemical reduction in H_3_BO_3_ solution. This procedure is referred to as indirect reduction (IR). Alternatively, the NP were directly obtained from a solution containing both CoSO_4_ and H_3_BO_3_, a procedure termed as direct reduction (DR). The electrochemical experiments were performed in a conventional three-electrode cell equipped with a platinum counter-electrode, a reference saturated calomel electrode (SCE), and the working electrode. The potential of the working electrode was controlled using a three-electrode potentiostat (Model CHI 660A electrochemical workstation, CH Instruments Inc., Austin, TX, USA). The synthesis conditions for all the samples are given in Table 1.

The effect of diazonium salt concentration and electrochemical reduction time on grafted organic layers was assessed by performing ex-situ spectroscopy using X-ray photoemission spectroscope (XPS) (Leybold-Heraeus LHS 560 spectrometer, Leybold France S.A., Villebon-sur-Yvette, France) and Mg Kα radiation. The Nafion film thickness was determined in terms of average step height (ASH) using a contact profilometer (DEKTAK 8, Bruker France S.A.S., Palaiseau, France). The dispersion and particle size analysis was carried out using a Multimode Nanoscope IIIa atomic force microscope (Bruker) operating in air. The AFM was operated using a silicon tip (OTESPA, Bruker AFM Probes, Camarillo, CA, USA) in the tapping mode in order to obtain high resolution topographic images and to avoid the issues of lateral forces and drag across the surface (in case of contact mode operation) that may otherwise damage the soft polymer surface through scratching by the sharp tip. Transmission electron microscopy (TEM) was performed using a Hitachi HNAR9000 microscope (Hitachi Ltd., Tokyo, Japan) (accelerating voltage: 300 kV, LaB_6_ point to point resolution: 0.18 nm). For TEM measurements, the nanoparticles were examined either by scratching the nanoparticle-supporting Nafion film onto the grid or via synthesis of Nafion supported nanoparticles on gold grids directly. In the latter case, the grid was attached to the modified Si surface followed by Nafion film deposition and subsequent metal ion reduction in the electrochemical cell. The grids were then peeled off from the supporting substrate.

The catalytic effect of nanoparticles was assessed by growing carbon nanostructures at 900 °C using the low-pressure microwave plasma-enhanced chemical vapor deposition (LP-PECVD) technique. The experimental setup is described elsewhere [48]. The values of applied power and the substrate bias were maintained at 125 W and −30 V, respectively. The total chamber pressure was 1.5 mTorr and the acetylene (C_2_H_2_) to ammonia (NH_3_) flow ratio was kept at 1:5.3 (sccm) for ~30 min.

## 4. Conclusions

Electrochemical process conditions can be tuned to obtain cobalt nanoparticles with desirable size and uniform distribution over Nafion films. Initially, surface passivation of the silicon wafer was accomplished through electrochemical grafting of the aryl group using para-nitrobenzene diazonium (PNBD) salt via chronoamperometry at −1.2 V for 150 ms. For Nafion film deposition, spin coating process produced more smooth films with minimum defect-density, as compared to the dip coating process.

Cobalt nanoparticles with average size from few tens of nanometers to <10 nm and bimodal or monodisperse size distribution can be produced through appropriate choice of electrochemistry parameters including metal precursor salt concentration, EC reduction time, and direct or indirect (ion uptake followed by reduction) EC reduction. Ultrafine cobalt nanoparticles with an average size of 2.8 ± 0.4 and 9.8 ± 2.6 nm, with some tendency of air oxidation, as indicated by EDS and HR-TEM studies, were produced using a 1 mM CoSO_4_ and 0.5 M H_3_BO_3_ mixed solution upon EC reduction for 5 and 20 s, respectively.

Vertically oriented carbon nanostructures with nanotubes and/or nanosheets morphology can be produced over catalyst NP supporting substrates through PECVD technique. When the NP size was decreased from several tens of nanometer to few nanometers, NP/CNT hybrid structures were obtained with the following characteristics: (a) large nanoparticles (>250 nm) along with few-walled CNT in case of bimodal NP size distribution, (b) relatively smaller (~120 nm) NP and multiwalled CNT with an outside diameter of 19 nm, and (c) clusters of few-walled carbon nanotubes and carbon nanosheets. An associated shift in the growth mechanism from tip- to base-growth was observed upon reduction in the CNT outside diameter. Such metal NP/carbon nanostructures hybrid compositions have potential applications in the areas of catalysis, supercapacitors, and sensors.

## Figures and Tables

**Table 1:** Sample identification scheme and the processing conditions during each step.

Sample ID	HF Etch ^a^	Grafting ^b^	Nafion Deposition Dip/Spin Coating	NP Synthesis IR/DR ^c^	*t*^d^, nm	NP Size nm
	Si	Si/PNBD	Si/PNBD/Naf	Si/PNBD/Naf/NP		
S_1_	√	√	×	×		
S_2_	√	√	0.5 wt %; Dip	IR 0.1 M CoSO_4_ (3 h); 0.5 M H_3_BO_3_ (−1.5 V, 5 s)	94 ± 5.7	5.4 ± 1.6
S_3_	√	√	0.5 wt %; Spin 4000 rpm 2 min	IR 0.1M CoSO_4_ (3h); 0.5 M H_3_BO_3_ (−1.5 V, 5 s)	180 ± 11	4.8 ± 2.0
S_4_	√	√	1.0 wt %; Spin 4000 rpm 2 min	IR 0.1 M CoSO_4_ (3 h); 0.5 M H_3_BO_3_ (−1.5 V, 20 s)	87 ± 5.1	128 ± 12 5.3 ± 1.1
S_5_	√	√	0.5 wt %; Spin 4000 rpm 2 min	DR 0.1 M CoSO_4_ 0.5 M H_3_BO_3_; (−1.5 V, 20 s)		65.2 ± 8.4
S_6_	√	√	1.0 wt %; Spin 4000 rpm 2 min	DR 1mM CoSO_4_ 0.5 M H_3_BO_3_; (−1.5 V, 5 s)		2.8 ± 0.4
S_7_	√	√	1.0 wt %; Spin 4000 rpm 2 min	DR 1 mM CoSO_4_ 0.5 M H_3_BO_3_; (−1.5 V, 20 s)		9.8 ± 2.6

^a^ HF 1 wt %, 5 min; ^b^ 5 mM PNBD 10 mM H_2_SO_4_, −1.2 V, 0.15 s; ^c^ CoSO_4_ Immersion + electrodeposition (Indirect Reduction, IR); Direct Reduction (DR); ^d^ *t* Nafion film thickness.

## Supplementary Materials

The following are available online at http://www.mdpi.com/1996-1944/11/5/687/s1, Figure S1: Average step height for the different samples (Table 1) indicating Nafion film thickness after dip coating or spin coating process, Figure S2: AFM area scans for the S_4_ and S_5_ samples (Table 1) indicating certain degree of agglomeration among the nanoparticles and bimodal size distribution in S_4_, Figure S3: Surface microstructure of the Co NP/Carbon nanostructures with EDS analysis and elemental maps for Co and C. (S_4_, Table 1).
