# Supplementary material for: Metal/Carbon Hybrid Nanostructures Produced from Plasma-Enhanced Chemical Vapor Deposition over Nafion-Supported Electrochemically Deposited Cobalt Nanoparticles
Source: Materials (Basel). 2018 Apr 27;11(5):687. doi: 10.3390/ma11050687 (PMC5978064; doi:10.3390/ma11050687)
Supplement: Supplementary file 1 [file materials-11-00687-s001.docx]

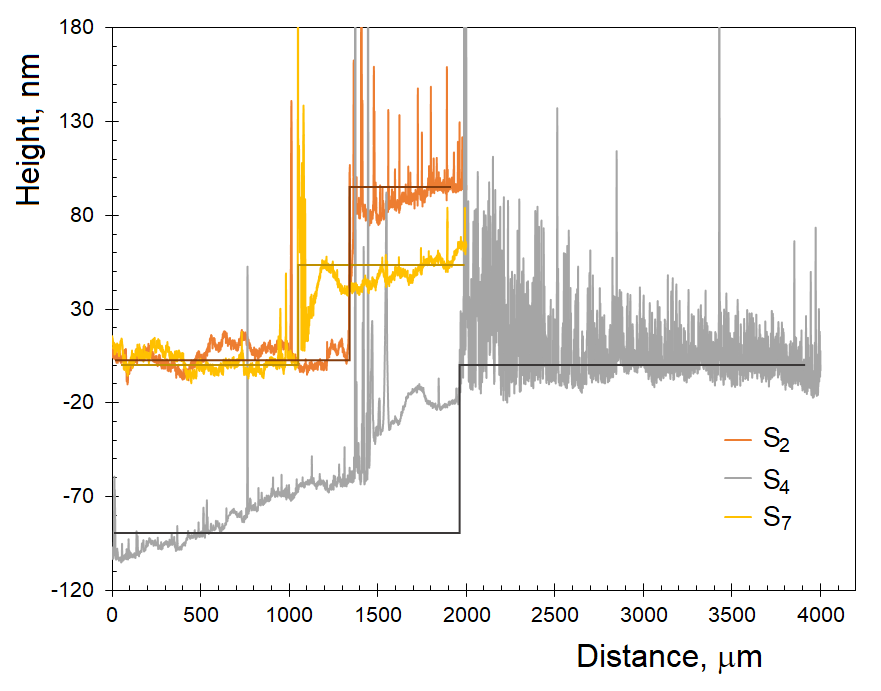


**Figure S1.** Average step height for the different samples (Table 1) indicating Nafion film thickness after dip coating or spin coating process.


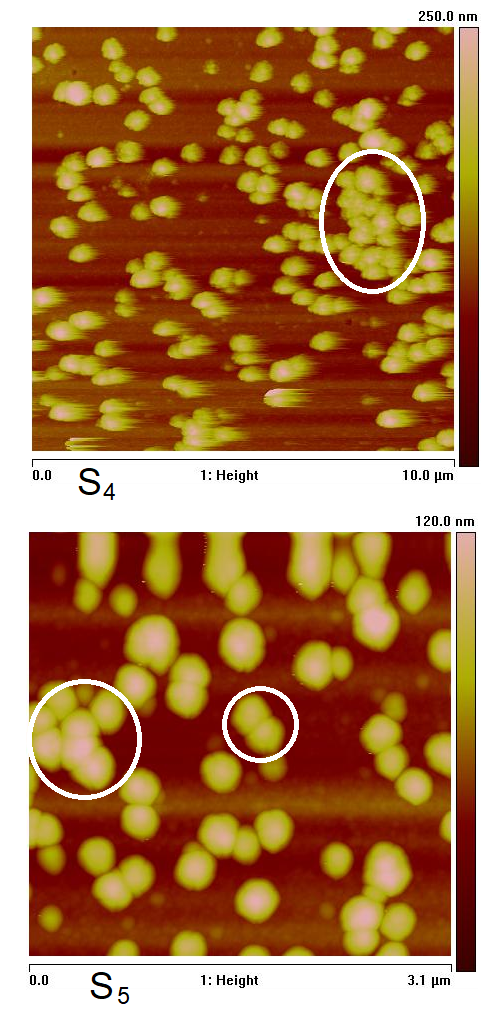


**Figure S2.** AFM area scans for the S_4_ and S_5_ samples (Table 1) indicating certain degree of agglomeration among the nanoparticles and bimodal size distribution in S_4_.


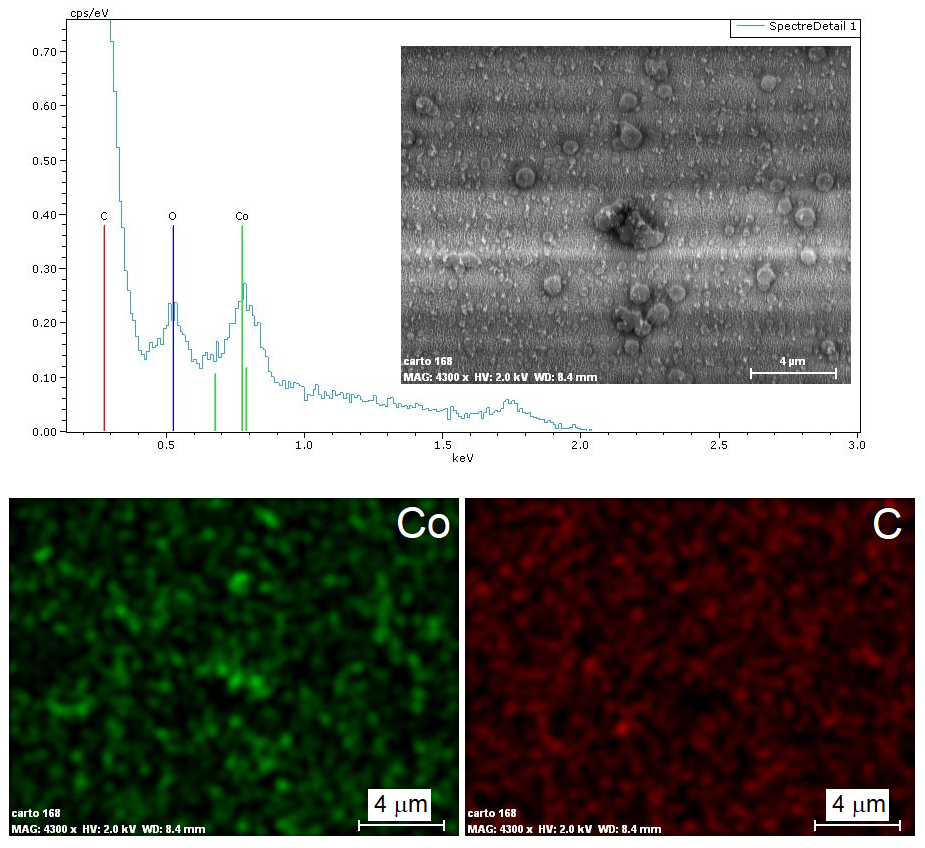


**Figure S3.** Surface microstructure of the Co NP / Carbon nanostructures with EDS analysis and elemental maps for Co and C. (S_4_, Table 1).
